# Magnetic Resonance Imaging–Guided Focused Ultrasound Positioning System for Preclinical Studies in Small Animals

**DOI:** 10.1002/jum.15514

**Published:** 2020-10-08

**Authors:** Theoharis Drakos, Marinos Giannakou, Georgios Menikou, Christakis Damianou

**Affiliations:** ^1^ Medsonic Ltd Limassol Cyprus; ^2^ Department of Electrical Engineering Cyprus University of Technology Limassol Cyprus

**Keywords:** magnetic resonance imaging, mouse, positioning, ultrasound

## Abstract

**Objectives:**

A positioning device compatible with magnetic resonance imaging (MRI) used for preclinical studies in small animals was developed that fits in MRI scanners up to 7 T. The positioning device was designed with two computer‐controlled linear stages.

**Methods:**

The positioning device was evaluated in an agar‐based phantom, which mimics soft tissues, and in a rabbit. Experiments with this positioning device were performed in an MRI system using the agar‐based phantom. The transducer used had a diameter of 50 mm, operated at 0.5 MHz, and focused energy at 60 mm.

**Results:**

Magnetic resonance thermometry was used to assess the functionality of the device, which showed adequate deposition of thermal energy and sufficient positional accuracy in all axes.

**Conclusions:**

The proposed system fits in MRI scanners up to 7 T. Because of the size of the positioning device, at the moment, it can be used to perform preclinical studies on small animals such as mice, rats, and rabbits.

**Abbreviations**
FUS: focused ultrasound
MR: magnetic resonance
MRgFUS: magnetic resonance–guided focused ultrasound
MRI: magnetic resonance imaging
US: ultrasound

Magnetic resonance–guided focused ultrasound (MRgFUS) was explored in 1993 by Hynynen et al.^1^ In that first report, it was shown that focused ultrasound (FUS) treatments could be monitored safely and adequately by magnetic resonance imaging (MRI) and with excellent contrast between necrotic tissue and normal tissue.

This interesting result motivated researchers to develop positioning devices to navigate ultrasound (US) transducers. The first robotic systems used hydraulic transmission means (in which tubes filled with pressurized water in tubes were used) to move the mechanical components of the system.^2^, ^3^, ^4^ However, hydraulic robotic systems had serious accuracy problems, and as a result, they were eventually abandoned.

The Israeli company InSightec (Tirat Carmel, Israel) produced the first commercial MRgFUS robotic system. Motion in various stages of the InSightec system was powered by piezoelectric motors. The original system focused on the treatment of uterine fibroids^5^ and adenomyosis.^6^ In 2004, the InSightec system obtained approval from the United States Food and Drug Administration. The InSightec device was upgraded in the following years to serve a variety of other applications ranging from prostate cancer treatment,^7^ hepatic cancer,^8^ breast cancer,^9^ and pain palliation of bone metastases,^10^, ^11^ and it was also deployed for essential tremor treatment.^12^

Philips Healthcare (Best, the Netherlands) also joined the area of MRgFUS^13^ by developing the commercial device Sonalleve. The Sonalleve device introduced 3‐dimensional cartesian positioning combined with two rotation angles, with the high‐intensity focused US transducer embedded in the MRI's patient table. The aforementioned system received a Conformité Européene mark for palliative MRgFUS applications in fibroid and bone metastases.

Thus, the technology of MRgFUS has matured by now, since commercial products are available for many applications. Therefore, there is a demand for exploring new applications in the area of MRgFUS. The new applications require experimentation in animal models. Thus, there is a great demand for positioning devices for preclinical studies. One of the first positioning devices was reported by Chopra et al,^14^ who developed an MRI‐compatible 3‐axis robotic FUS system for small animals. Later, FUS instrument commercialized this device for use in small animals.^15^

The European company Medsonic developed several robotic systems for animal use. One such system was developed for use in rabbit brains.^16^, ^17^ Another system was dedicated to preclinical use of prostate MRgFUS^18^, ^19^ using a transrectal US transducer. These systems combine linear and angular stages for setting the current position of the transducer, and both can be placed on top of the MRI table for sliding along with the subject in the scanner's bore.^4^, ^19^

The French company Image Guided Therapy (Pessac, France)^20^ also developed a dedicated MRgFUS system for companion animals. Finally, InnoMotion (InnoMedic GmbH, Herxheim, Germany) developed a positioning device that can be used to hold a FUS transducer. The first version of the InnoMotion system was designed for performing needle biopsies under MRI guidance^21^ with 5 degrees of freedom of pneumatically driven stages.

This article describes an updated version of a small‐animal positioning device for conducting preclinical studies of MRgFUS applications. The previous positioning device developed by our group^22^ was also dedicated to small animals, but its size was such that it could only fit in 1.5‐ or 3‐T MRI systems. The proposed system is reduced in size to fit in MRI scanners up to 7 T.

The proposed positioning device may carry a single‐element transducer up to a diameter of 100 mm. Based on the size of the robot and targeted animals, the maximum radius of curvature that can be used with this system is 60 mm. Due to the fact that small animals will be used, there is no need for an x‐axis. Therefore, two linear stages are needed (z and y). Because of the size of small animals, there is no need to move in the x‐axis. An angular axis might be needed for some applications even for small‐animal applications, but to keep the system simple, an angular axis was not incorporated in this device. It is possible to use an angular axis in future designs. The target of the design is to offer an alternative and affordable solution to end users rather than replacing the phased array systems that already exist. Among the advantages of the proposed system compared to the conventional phased array systems is the lack of design complexity and the decreased production cost. The designed system can be applied in multiple small‐animal applications, and because of the small size of the subjects, it is possible to achieve transcranial penetration using a single‐element transducer. Marketing‐wise, transcranial applications in small animals are the most attractive ones, since equivalent interventional procedures are complex and are hardly ever attempted. Driving the transducer with a robotic positioner increases the translation access range to the target, offering improved efficacy and safety of the treatment procedure.

## Materials and Methods

The device and US protocols were evaluated with tissue‐mimicking, agar‐based phantoms. No animals or patient data were included in this study. Therefore, no informed consent from patients or approval from an Ethics Committee was required.

### Mechanical Design of the Positioning Device

**Figure 1:**
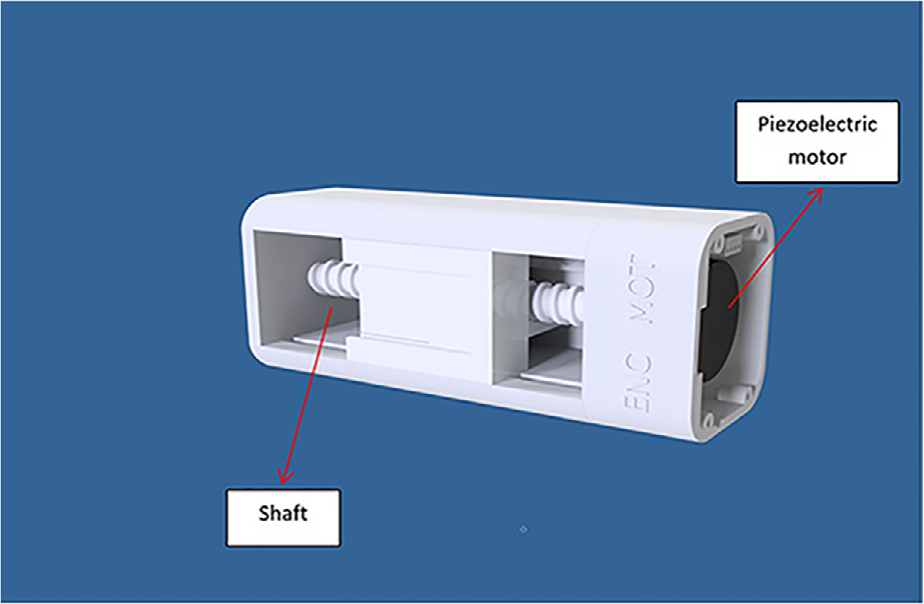
Computer‐aided design drawing of the linear axis for motion along the MRI y‐axis.

**Figure 2:**
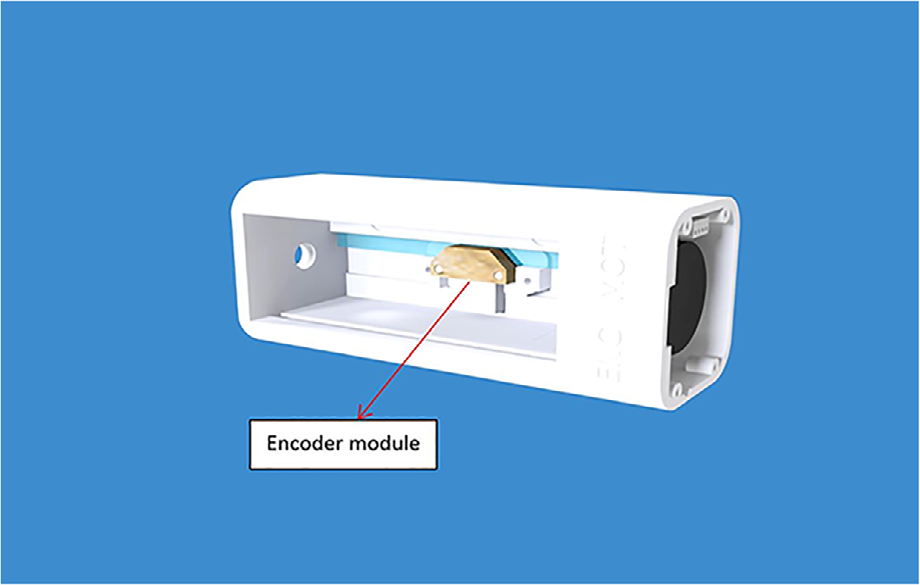
Placement of the encoder modules in one of the linear stages.

**Figure 3:**
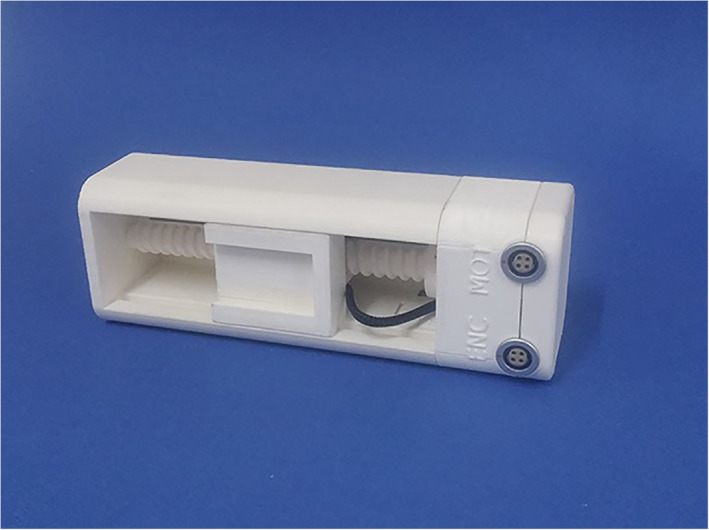
Developed y‐axis stage.

The device includes two computer‐controlled axes (z and y on MRI). Figure 1 shows a drawing of the linear axis for motion along the MRI y‐axis. The y‐plate was coupled to a threaded plastic screw, which was attached to the shaft of a piezoelectric motor (USR 30‐S3; Shinsei Kogyo Corp, Tokyo, Japan). The rotation of this shaft converts the angular motion to linear. An optical encoder module (US Digital Corporation, Vancouver, WA) was attached under the y‐axis frame to record the motion of the encoder strip. The encoder module (EM1‐0‐500‐I; US Digital Corporation) was used for both stages. The encoder output is wired to the counter pin of a data acquisition board (6251; National instruments, Austin, TX). Figure 2 shows the position of the transmissive optical encoder module for controlling linear motion. A photograph of the finalized y‐stage motion component is shown in Figure 3.

**Figure 4:**
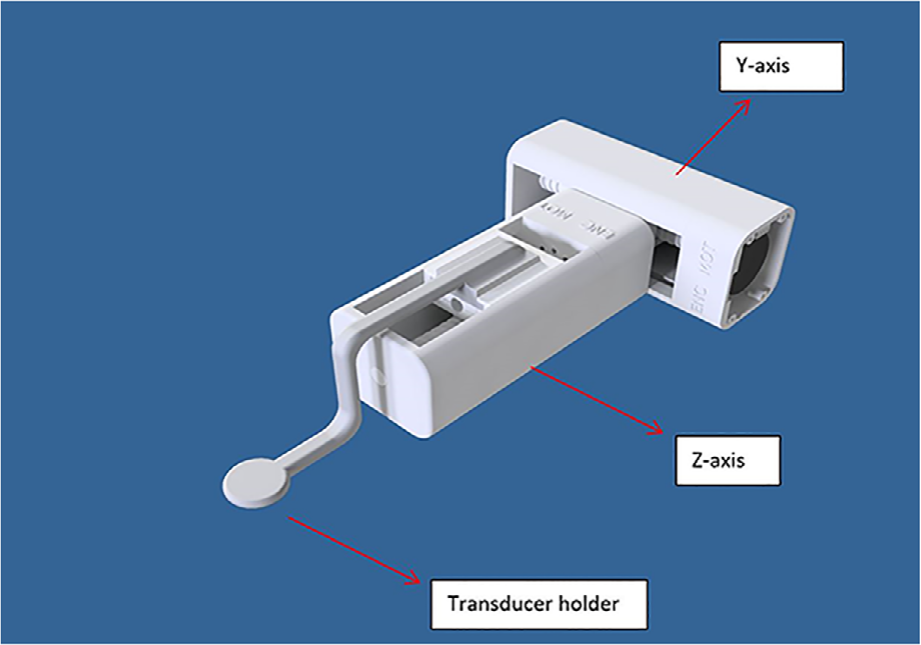
Coupling of the two linear stages (z‐axis stage and y‐axis stage). The transducer arm was coupled to the z‐axis plate.

Compared to our previous small‐animal positioning device,^22^ the main difference is the size of the motor, which is smaller in the current device (almost 50% smaller). This enabled us to design a smaller positioning device. The z‐axis stage was coupled to the y‐axis with a simple structure, as shown in Figure 4. The principle of movement of the z‐stage and its range were the same as for the y‐stage. The placement of the encoder followed the same technique as the y‐stage. The transducer arm was coupled to the z‐axis plate, as shown in Figure 4. Acoustic coupling was established by immersing the transducer in a tank with degassed water.

A 3‐dimensional computer‐aided model of the positioning device was designed in MicroStation version 8 (Bentley Systems, Inc, Exton, PA), which was later fabricated in acrylonitrile butadiene styrene material by using fusion deposition modeling technology (FDM400; Stratasys, Eden Prairie, MN).

**Figure 5:**
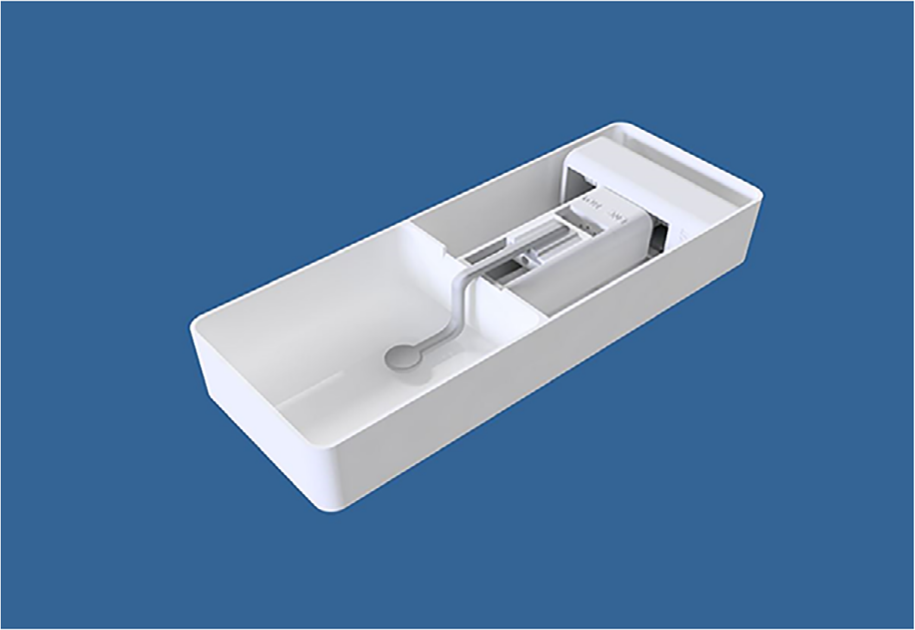
Interior of the positioning device.

**Figure 6:**
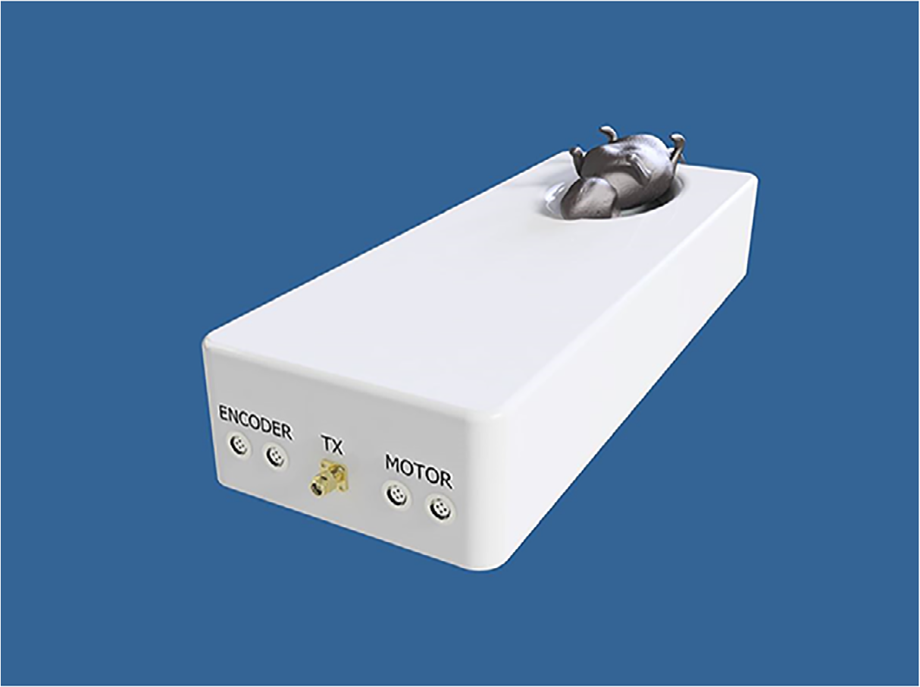
Complete robotic system showing both linear axes.

The positioning device can be placed on the table of any commercial MRI scanner except a 9.4‐T scanner. The dimensions of the device with all axes extended do not exceed greater than 7, 40, and 15 cm in height, length, and width, respectively. The motion range of the robot is 6 for both axes. The positioning device weighs around 2.3 kg. Figure 5 shows the interior of the device, and Figure 6 shows the entire device.

### Mouse Holder

**Figure 7:**
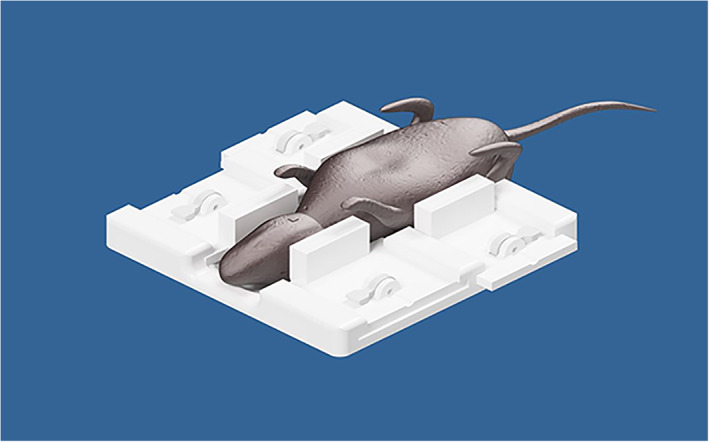
Computer‐aided design drawing of the mouse holder.

**Figure 8:**
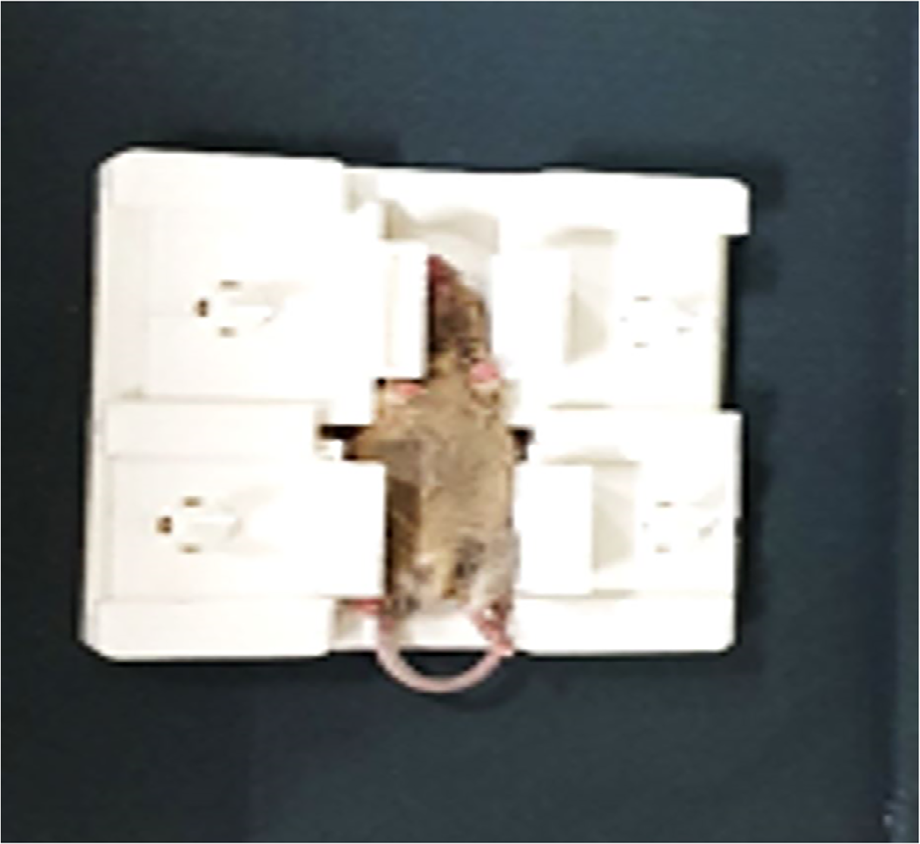
Mouse holder.

To prevent motion of the mouse, a specially designed holder was developed. When the mouse is positioned in the supine position, sideways movable holders are pushed toward the mouse, preventing any mouse movement (Figure 7). Figure 8 shows a mouse placed in the experimental setting.

### Software

An application was developed using the C# programming language (Visual Studio 2010 Express; Microsoft Corporation, Redmond, WA), which aimed to enhance the user interface. The two motion axes of the device are controlled by selecting an automated algorithm^23^ or by specifying a direction and step to move. The software includes additional functions, such as an interface with the MRI, magnetic resonance (MR) thermometry, and US control (eg, frequency, power, and sonication time).

### Electronics

The metallic housing containing the positioning device's motor drivers was kept outside the Faraday cage to eliminate the possibility of electromagnetic interference. Each US motor is driven by its corresponding driver (D6030; Shinsei Kogyo Corp). The drivers are powered with 24 V, which is provided by a DC power supply. A universal serial bus data acquisition board (6251; National Instruments) was wired to the drivers to rotate the motors in a clockwise or anticlockwise direction based on instructions from the software.

### High‐Intensity Focused US System

The high‐intensity focused US system includes a signal generator (HP 33120A; Agilent Technologies, Englewood, CO), a radiofrequency amplifier (AG1012; T & C Power Conversion, Inc, Rochester, NY), and a 50‐mm‐diameter spherical transducer (Medsonic Ltd, Limassol, Cyprus) operating at 0.5 MHz with an 80‐mm focal length. The transducer consists of a type P762 piezoceramic active element (Ferroperm; Kvistgaards, Denmark) sealed with a thermoresistant epoxy backing material for damping excessive vibrations. The impedance of the transducer was matched to 50 Ω. The transmission of high‐frequency harmonics was reduced by connecting a custom‐made low‐pass filter with a cutoff frequency of 10 MHz in series.

### Tissue‐Mimicking, Agar‐Based Phantom

The performance of the high‐intensity FUS transducer was assessed through a series of sonications using a tissue‐mimicking, agar‐based phantom. The phantom was fabricated by following a recipe that was developed and characterized previously for its properties by our group.^24^, ^25^ Magnetic resonance thermometry was used to monitor the temperature–time profile and deposition of heat in the phantom during sonications. The efficacy of the exposure protocols was assessed by using the produced color‐coded thermal maps.

### Animal Testing

The system was evaluated in one New Zealand rabbit that was transferred to the laboratory from an approved farm.

### Magnetic Resonance Imaging

The positioning device system was tested in a 1.5‐T MR system (Signa 1.5 T; GE Healthcare, Fairfield, CT). Magnetic resonance imaging with a high resolution was conducted to visualize the phantom/transducer setup. A T2‐weighted fast spin echo sequence was used with the following parameters: repetition time, 2500 milliseconds; echo time, 60 milliseconds; slice thickness, 3 mm; matrix, 256 × 256; field of view, 16 cm; number of excitations, 3; and echo train length, 8. A T1‐weighted spoiled gradient sequence was used for acquiring MR thermometry: repetition time, 50 milliseconds; echo time, 2.7 milliseconds; field of view, 16 cm; matrix, 256 × 256; flip angle, 30°; and number of excitations, 1.

### Magnetic Resonance Thermometry

The temperature during the high‐intensity focused US protocol was estimated by the proton resonance frequency shift equation reported first by Ishihara et al.^26^ The equation corelates the temperature elevation (Δ*T*) with the measured phase as follows:

$$\mathrm{\Delta T}=\frac{\varphi \left(T\right)-\varphi \left(T_{0}\right)}{\gamma \alpha {Β}_{0}\mathrm{TΕ}},$$

where *φ*(*T*) and *φ*(*T*_0_) are the phases at an initial temperature (*T*) and at a final temperature (*T*_0_); *α* is the pulse repetition frequency change coefficient; *γ* is the gyromagnetic ratio; *B*_0_ is the magnetic field strength; and *TE* is the echo time. The spoiled gradient pulse sequence was used to acquire the MRI thermometric maps.

## Results

**Figure 9:**
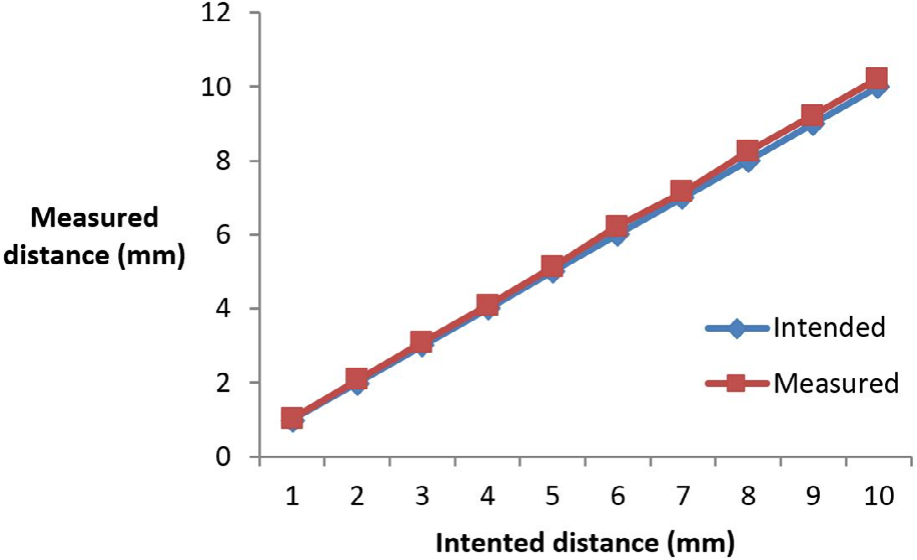
Measured linear step versus intended step in millimeters for the y‐axis.

The accuracy of the linear axis (y) was assessed comparing the intended step to the actual distance that the robot was moved. Figure 9 shows the difference of the measured distance to the intended distance. This procedure was repeated for step sizes of 1 to 10 mm. The values on the vertical axis represent the measured distance, which for each step was calculated by the average of 20 such measurements. The measured distance was 0.04 mm larger compared to the intended distance for a 1‐mm step, whereas for a 10‐mm step, the difference was 0.1 mm larger compared to the intended step. Similar differences were observed for the z‐axis. The smallest step that could be achieved in any linear axis was 0.1 mm.

**Figure 10:**
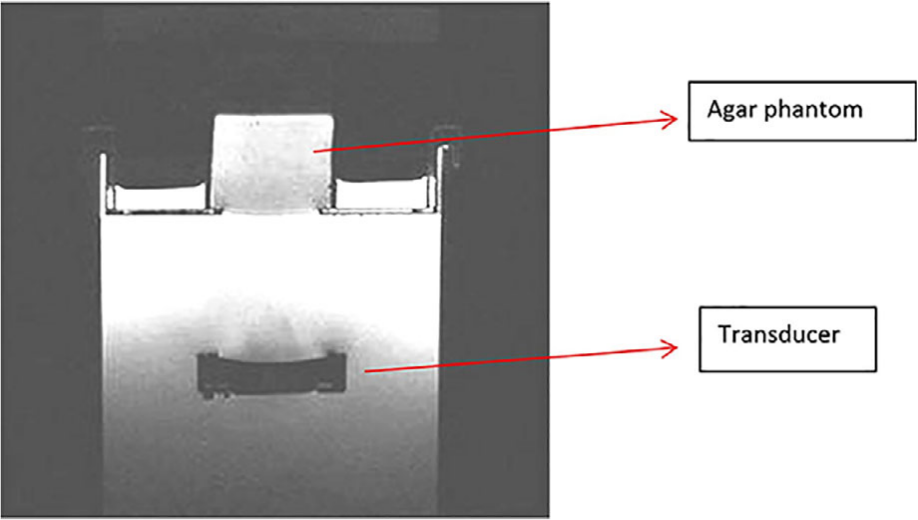
T2‐weighted fast spin echo MRI of the transducer of the positioning device and the agar phantom that was used to produce MR thermometry.

The next series of figures were obtained in the MRI setting. Figure 10 shows a T2‐weighted fast spin echo MR image of the transducer of the positioning device and the agar phantom that was used to produce MR thermometry. No MRI compatibility evaluation was performed, since the same motors and encoders were used in previous studies by our group.^23^, ^27^, ^28^, ^29^ Note the excellent contrast between water, the agar‐based phantom, and the transducer. The images did not reveal any air spaces between the water and agar phantom interface. The image coil was placed around the agar‐based phantom.

**Figure 11:**
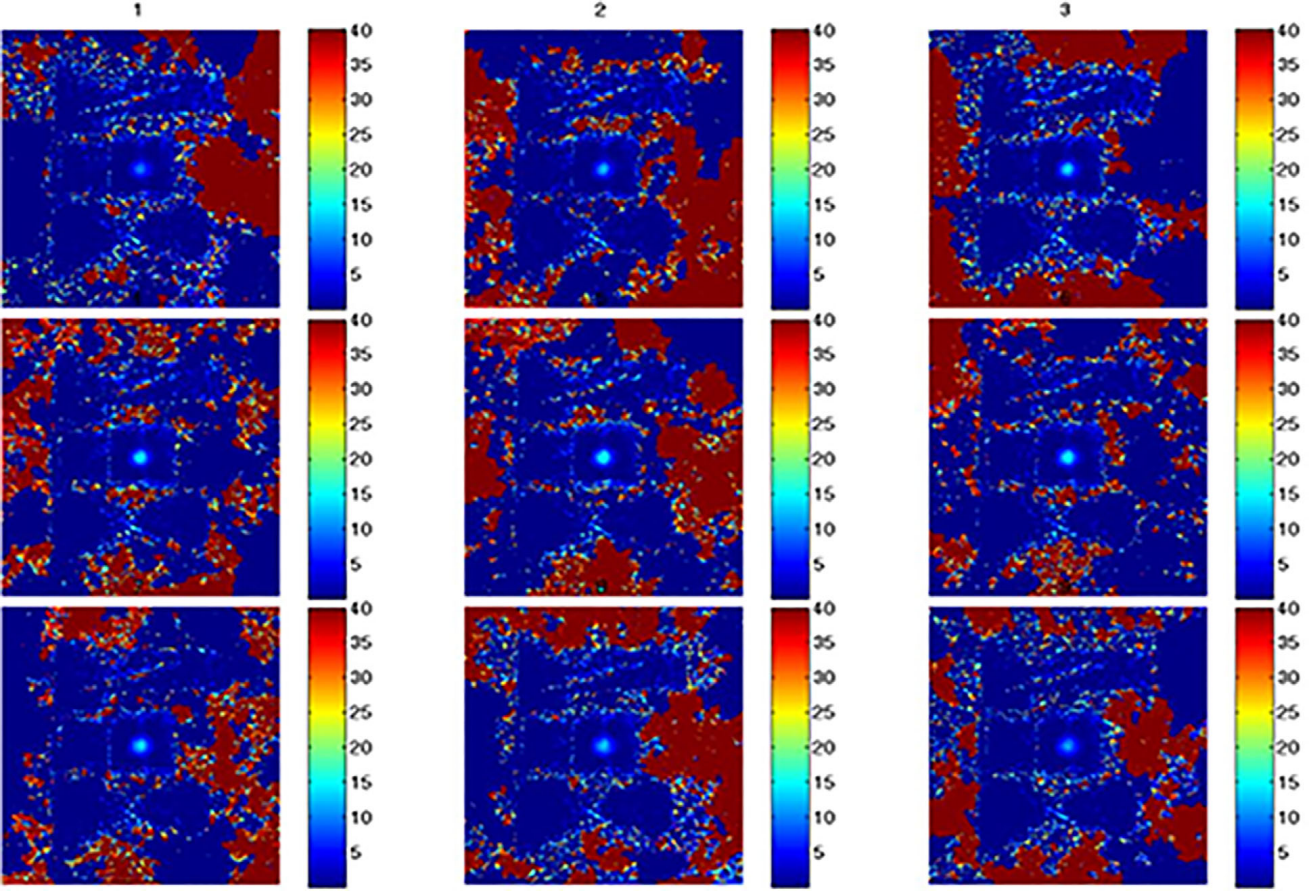
Magnetic resonance thermometric map in a coronal plane at different intervals (every 12 seconds) using acoustic power of 20 W for 60 seconds.

**Figure 12:**
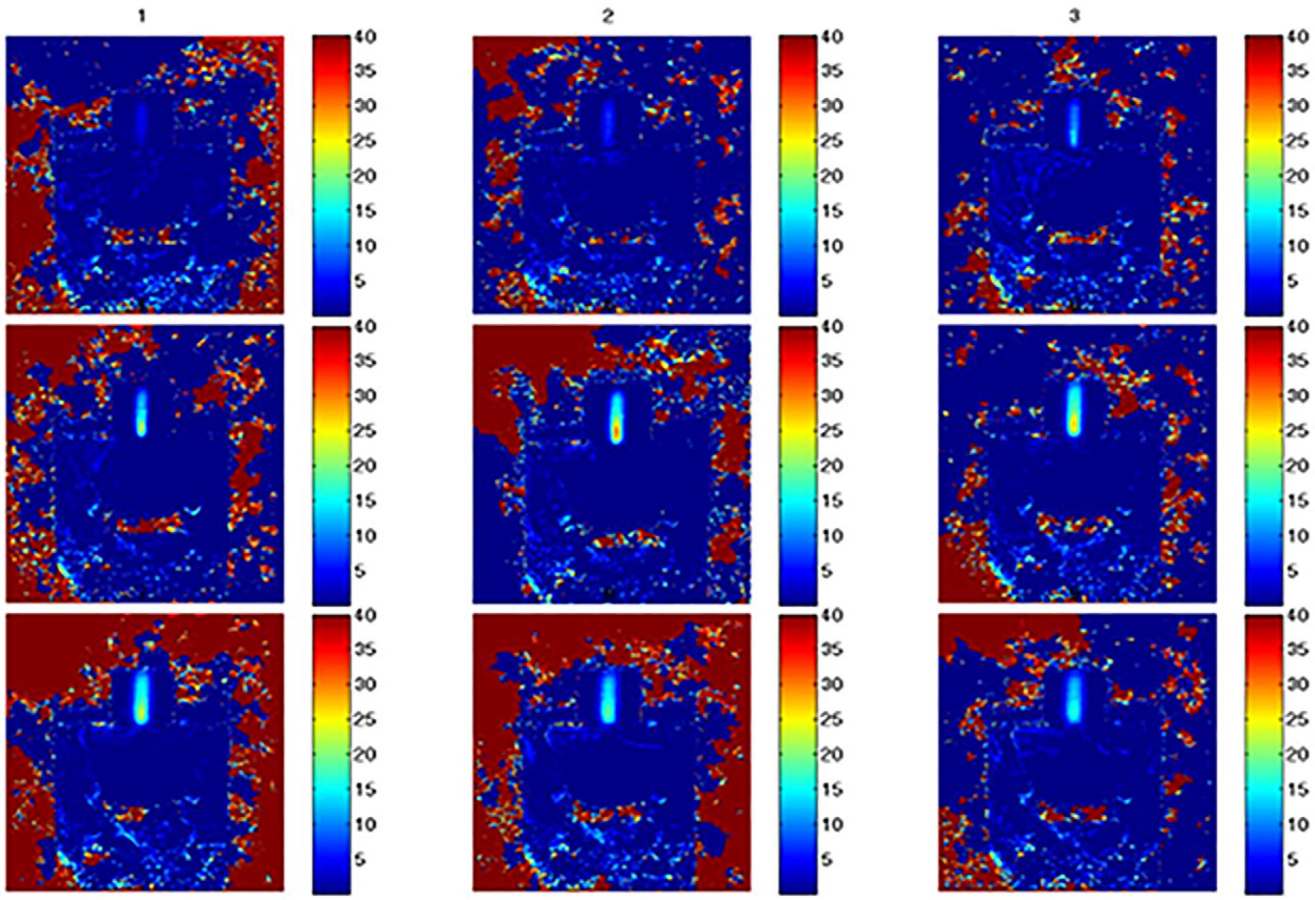
Magnetic resonance thermometric map in a sagittal plane at different intervals (every 12 seconds) using acoustic power of 20 W for 60 seconds.

A time series of MR thermometry in the coronal plane with a 12‐second temporal resolution used to monitor heat deposition in the phantom induced by a 20‐W, 60‐second sonication is shown in Figure 11. Figure 12 shows the corresponding MR thermometric time series in the sagittal plane, showing heat build‐up induced by the proposed transducer.

**Figure 13:**
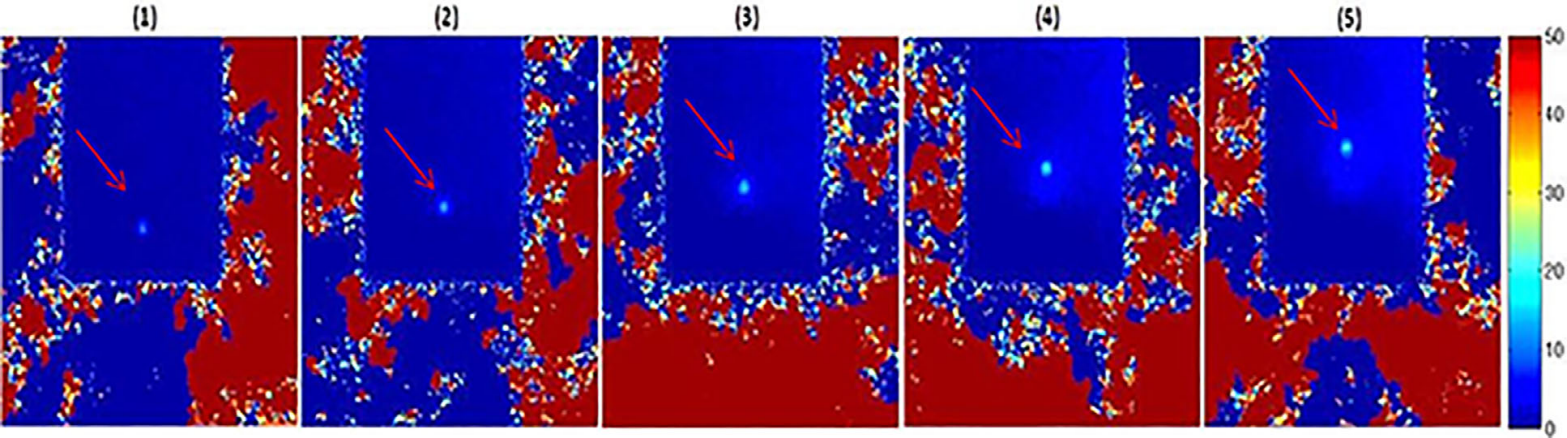
Magnetic resonance thermometric map in a coronal plane showing the motion of one of the linear stages of the positioning devices. The acoustic power used was 20 W for 60 seconds.

Figure 13 shows an MR thermometric map in a coronal plane, showing the motion of one of the linear stages of the positioning devices. Heating was induced at each step via 20‐W acoustic power sonications applied for 20 seconds. The intended spatial step was 10 mm. The average distance measured by MR thermometry was 9.95 mm with an SD of 0.2 mm (n = 12).

**Figure 14:**
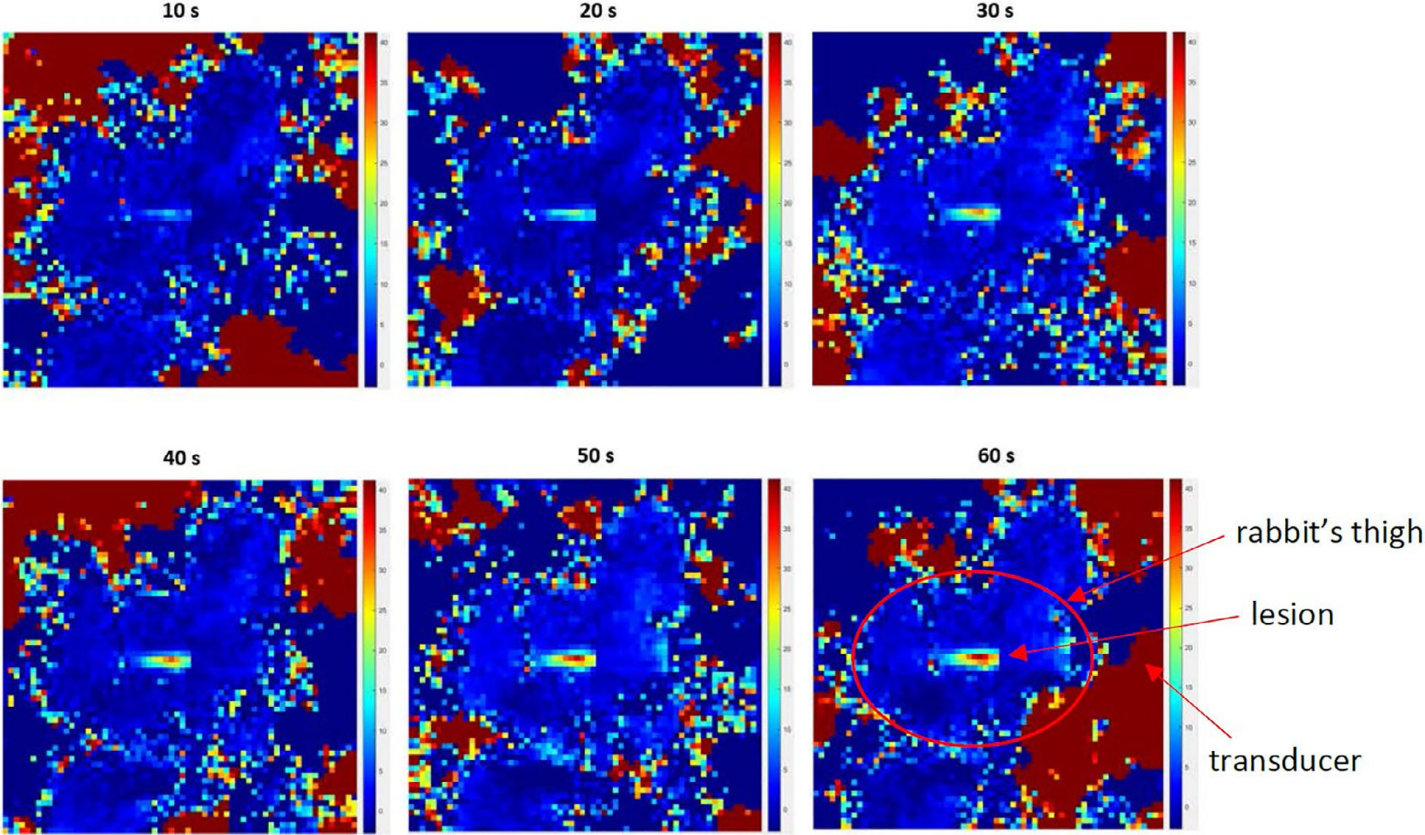
Temperature map produced in the thigh muscle of a rabbit. The power used was 27 W for 60 seconds. The temperature map is in a plane parallel to the transducer propagation.

Figure 14 shows a temperature map produced in the thigh muscle of a rabbit. The power used was 27 W for 60 seconds. The temperature map is in a plane parallel to the transducer propagation. Despite the small dimensions of the thigh, it was possible to place the beam in the center of the thigh muscle.

## Discussion

An MRgFUS positioning device was developed for use with small animals (mice, rats, and rabbits), which can fit in commercial MRI scanners up to 7 T with a bore diameter of 30 cm or larger. The motion accuracy of the positioning device was tested with MR thermometry by using 10‐mm steps. The measured distance was compared to the intended distance, resulting in excellent spatial accuracy. The tests were performed in a custom‐made agar/silica/evaporated milk gel phantom.

The positioning device can displace the transducer in the z‐ and y‐axes with adequate accuracy; therefore, a predefined grid of thermal lesions can be delivered. The system has the ability to image and move at the same time because of the use of piezoelectric motors and optical encoders that are MRI compatible.

The proposed system displays a 6‐cm range of motion in both axes, which offers sufficient coverage for most applications. The design of this robot can be scaled up so that it can be applied in humans. It can be extended to greater than 15 cm in both axes and therefore can be used for 1.5‐ or 3‐T MRI scanners. The positioning device is 7 cm in height. With an improved design, this can be reduced even further. With the above‐mentioned adjustments to the design, the proposed system can enter the market for human use in the abdominal area (liver, kidney, and pancreas), fibroids, or the breast. The expansion to a human positioning device will require the addition of one computer‐controlled linear axis and two computer‐controlled angular axes.

The MR thermometry shown in Figures 10 and 11 demonstrated the heating capabilities of the low‐frequency transducer by using the pulse repetition frequency method. This type of transducer is suitable for transcranial sonication of mice.^30^ A thorough evaluation of the transducer's heating performance in small‐animal models is essential.

The accuracy of the linear‐displacement axis was not compromised in the vicinity of the MRI‐compatible encoders and was equal to 0.1 mm, which exceeded the requirements of interventional oncology applications. It is the group's target to expand the portfolio by designing and producing MRI‐compatible positioning devices specific to new FUS surgical applications. As mentioned elsewhere, the goal is to add additional axes (one linear and two angular) to use it in the clinical setting with improved maneuverability. The key advantages of the proposed positioning device are the low cost and simplicity, but it is functional and accurate device.

The main innovation of this device is that its size is small compared to other available devices. The compactness of the system improves handling of the anesthetized animal by leaving more free space for the associated accessories and immobilization pads. Additionally, the device can fit in MRI systems with bore sizes as small as 30 cm. Currently, the only device that can do this^20^ uses phased arrays, which steer the beam electronically. To our knowledge, there is another single‐element transducer system for small animals^31^ but with a completely different design. The design of the system is simpler yet as effective as phased arrays, and the production cost is affordable, since sonications are performed via a monoelement focused transducer. In our opinion, for experimental work in small animals, the use of a positioning device with two axes and the use of a single‐element transducer are sufficient.
